# Respiratory syncytial virus infection trend is associated with meteorological factors

**DOI:** 10.1038/s41598-020-67969-5

**Published:** 2020-07-02

**Authors:** Ilada Thongpan, Sompong Vongpunsawad, Yong Poovorawan

**Affiliations:** Center of Excellence in Clinical Virology, Faculty of Medicine, Chulalongkorn University, Bangkok, 10330 Thailand

**Keywords:** Viral epidemiology, Viral infection

## Abstract

Respiratory syncytial virus (RSV) infects young children and causes influenza-like illness. RSV circulation and prevalence differ among countries and climates. To better understand whether climate factors influence the seasonality of RSV in Thailand, we examined RSV data from children ≤ 5 years-old who presented with respiratory symptoms from January 2012–December 2018. From a total of 8,209 nasopharyngeal samples, 13.2% (1,082/8,209) was RSV-positive, of which 37.5% (406/1,082) were RSV-A and 36.4% (394/1,082) were RSV-B. The annual unimodal RSV activity from July–November overlaps with the rainy season. Association between meteorological data including monthly average temperature, relative humidity, rainfall, and wind speed for central Thailand and the incidence of RSV over 7-years was analyzed using Spearman’s rank and partial correlation. Multivariate time-series analysis with an autoregressive integrated moving average (ARIMA) model showed that RSV activity correlated positively with rainfall (r = 0.41) and relative humidity (r = 0.25), but negatively with mean temperature (r = − 0.27). The best-fitting ARIMA (1,0,0)(2,1,0)_12_ model suggests that peak RSV activity lags the hottest month of the year by 4 months. Our results enable possible prediction of RSV activity based on the climate and could help to anticipate the yearly upsurge of RSV in this region.

## Introduction

Respiratory syncytial virus (RSV) infection contributes to significant morbidity and mortality associated with lower respiratory tract infection*.* Illness associated with RSV infection is estimated to cause approximately 24.8 million episodes and 76,600 deaths annually^1^. Epidemiological studies have shown that infants, young children, and the elderly are especially at risk of RSV infection^2,3,4^. In regions with temperate climate, incidence of RSV infection concentrates during the winter months and may sometimes be absent in summertime^5^. In sub-tropical and tropical regions, RSV infection may be sporadic throughout the year or showed increased activity during the rainy or cooler months^6^.

The observed differences in RSV activities among countries located at similar latitudes from the equator, and cities within the same country, suggest that local weather pattern may partially play a role in dictating RSV seasonality. For example, a number of retrospective studies using epidemiological surveillance data in Brazil have shown different seasonal pattern for RSV attributable to the widely diverse regional climate characteristics there^7,8,9,10^. Meanwhile, some tropical countries have reported correlations between the frequency of RSV infection with peak rainfall and warmer temperatures^11,12,13,14,15^*.* It had been postulated that the increased rainfall and high temperatures were associated with the stability, survival, and virus transmission in the environment^16,17^. Some studies have shown that environmental data could enable the prediction of RSV epidemiology^18,19,20,21,22^, but it is not universally applicable since the seasons in which RSV epidemic occurs generally depend on geographic location and altitude^23^*.* There have been few studies examining RSV seasonality and weather pattern in Southeast Asia, none of which have been conducted in recent years^24^. In addition, findings in previous studies have at times been contradictory. Despite the shared geographical proximity, any slight differences in climate, socio-economy, cultural practices, and time of study among neighboring countries may influence observation outcome. Therefore, regional data are needed to most accurately forecast the onset or height of RSV activity in a given locality.

In the tropical climate of Thailand, RSV prevalence is historically highest during the rainy season, which is typically between May and November^4,25,26^. Thus far, there has been no studies examining any potential linkage between weather pattern and RSV infection cycle in Thailand. Forecasting the dynamics of RSV infection, which is responsible for a sizable portion of lower respiratory tract infection among infants and young children and for which no approved vaccines nor antivirals are available, could be useful towards evaluating the epidemiological burden of RSV in the region. Here, we analyzed the pattern of laboratory-confirmed RSV prevalence from 2012 to 2018 and variations in the climate factors including the amount of average rainfall, relative humidity, temperature, and wind speed by using correlation analysis. Using the time series models approach, which assumes that prevalence data from the past several years can predict future trends and captures any lagged relationships among variables, we retrospectively forecasted RSV activity and compared the model with the actual incidence. Knowledge of the impact of climate variables on RSV seasons in Thailand may be useful in predicting the magnitude of the annual RSV infection in the region and assist in the planning of healthcare resources to handle this important respiratory infection.

## Methods

### Study samples and ethics statement

This retrospective study used previously collated data from January 2012 to December 2018 involving the analysis of nasopharyngeal or throat swabs from three hospitals (King Chulalongkorn Memorial Hospital, Bangpakok 9 International Hospital, and Chum Phae Hospital) submitted for routine RSV testing (n = 8,209) (Table S1). Respiratory samples were from children 5 years of age or younger with influenza-like illness (defined as having temperature > 38 °C and respiratory tract symptoms such as runny nose, cough, sore throat, and difficulty breathing). From 2012 to 2015, RSV testing was done by using conventional reverse-transcription polymerases chain reaction (RT-PCR) assay^25^, while samples from 2016 onward were screened by using real-time RT-PCR and extended an earlier investigation of an ongoing RSV surveillance^4^. Informed consent was obtained from all subjects, and all methods were carried out in accordance with relevant guidelines and regulations. The Institutional Review Board of the Faculty of Medicine of Chulalongkorn University approved this study (IRB number 609/59).

### Climate data

Three seasons predominates in central Thailand. Summer (mid-February to mid-May) and rainy (mid-May to mid-October) seasons are followed by the relatively dry and cool months (mid-October to mid-February). We used the average monthly temperature (°C), rainfall (mm), relative humidity (%), and wind speed (m/s) data from VTBD (a location indicator designated by the International Civil Aviation Organization) weather station at the Thailand Bangkok Don Mueang International Airport (latitude 13.91°N and 100.6°E longitude). We also extracted the climate data from the Wolfram Alpha database (WolframAlpha 2019) (Table S2).

### Data analysis

We used the time series approach involving monthly data to examine the associations between RSV confirmed cases and climate variables. Relationships of RSV incidence with meteorological factors were evaluated using Spearman rank correlation. To remove collinearity among variables, partial correlations were evaluated. The RSV data set was plotted as a time series and assessed for stationarity using the augmented Dickey-Fuller test for unit roots. Multiple time series analysis was based on autoregressive integrated moving average (ARIMA) models. Prediction of the value of the target variable with a linear function of lag values (autoregressive) and an effect from recent random shock values (moving average) self-selected the most suitable ARIMA model for the influence of meteorological factors based on past incidence of RSV. ARIMA models have the flexibility to control the autocorrelation of time series data, and the Box-Ljung test was used to test the null hypothesis that the autocorrelations of the residual time series were equal to zero. An ARIMA model for the monthly incidence was then developed following the Box-Jenkins approach^27^.

To test the model-based prediction of RSV infections derived from meteorological data, another ARIMA model was used for the time period from January 2012 to December 2017 (referred to as the estimation period) and evaluated by comparing the predicted versus the observed incidence of RSV for the entire 2018 year. Using the ARIMA model based on the monthly number of RSV confirmed cases, we then examined the relative risk and lag effects in greater detail by fitting lag models that included relative humidity, rainfall, temperature, and wind speed terms at each lag period for as long as 4 months. Root mean square error (RMSE) was used to compare the prediction accuracy from each model, and the model which has the lowest RMSE was deemed as the best model. For statistical tests, P-values < 0.05 were considered statistically significant. All analyses were performed using R software, version 3.6.1 (https://www.R-project.org). Moreover, the sequence stationarity was tested by the augmented Dickey-Fuller (ADF) test using the R software, version 3.6.1, which enabled the use of untransformed time sequence monthly percentage of RSV from 2012 to 2018.

## Results

### RSV subgroups in circulation

**Figure 1:**
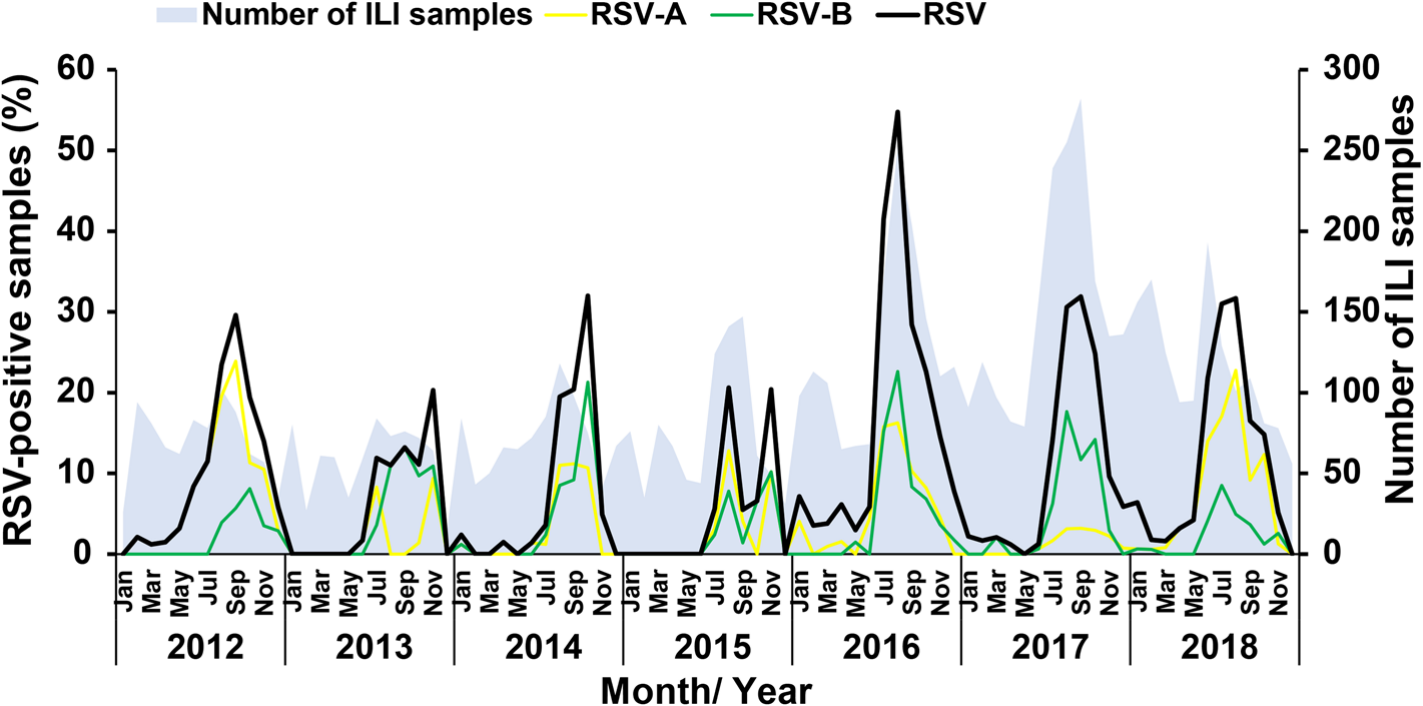
Seasonal distribution of RSV from 2012 to 2018. The number of ILI samples arriving each month (gray area, right Y-axis) is shown in comparison to the monthly percentage of all laboratory-confirmed RSV-positive samples (black line, left Y-axis). Upon typing, monthly percentage of RSV-A (yellow line) and RSV-B (green line) are also shown.

Respiratory samples were from children with the mean age of 2.45 years and a male-to-female ratio of 1 to 0.8. From January 2012 to December 2018, approximately one in ten samples (13.2%, 1,082/8,209) tested positive for RSV. Among RSV-positive samples, 46 children contributed two RSV-positive samples on two separate occasions (median time between infections, 13 months; range, 4–35 months) (Table S3). One child tested positive for RSV every year from 2016 to 2018. Most children who tested positive for RSV were one year of age or younger, and the average age of children with RSV infection did not appear to change from year to year (Fig. S1). The highest proportion of monthly RSV-positive samples appeared between July and November, which coincided with the local rainy season (Fig. 1). Although both RSV-A and RSV-B co-circulated during this time, RSV-A predominated in 2012 and 2018, while RSV-B was predominant in 2017. Both subgroups co-circulated equally from 2014 to 2016.

### Association of RSV with meteorological factors using bivariate and multivariate analyses

**Figure 2:**
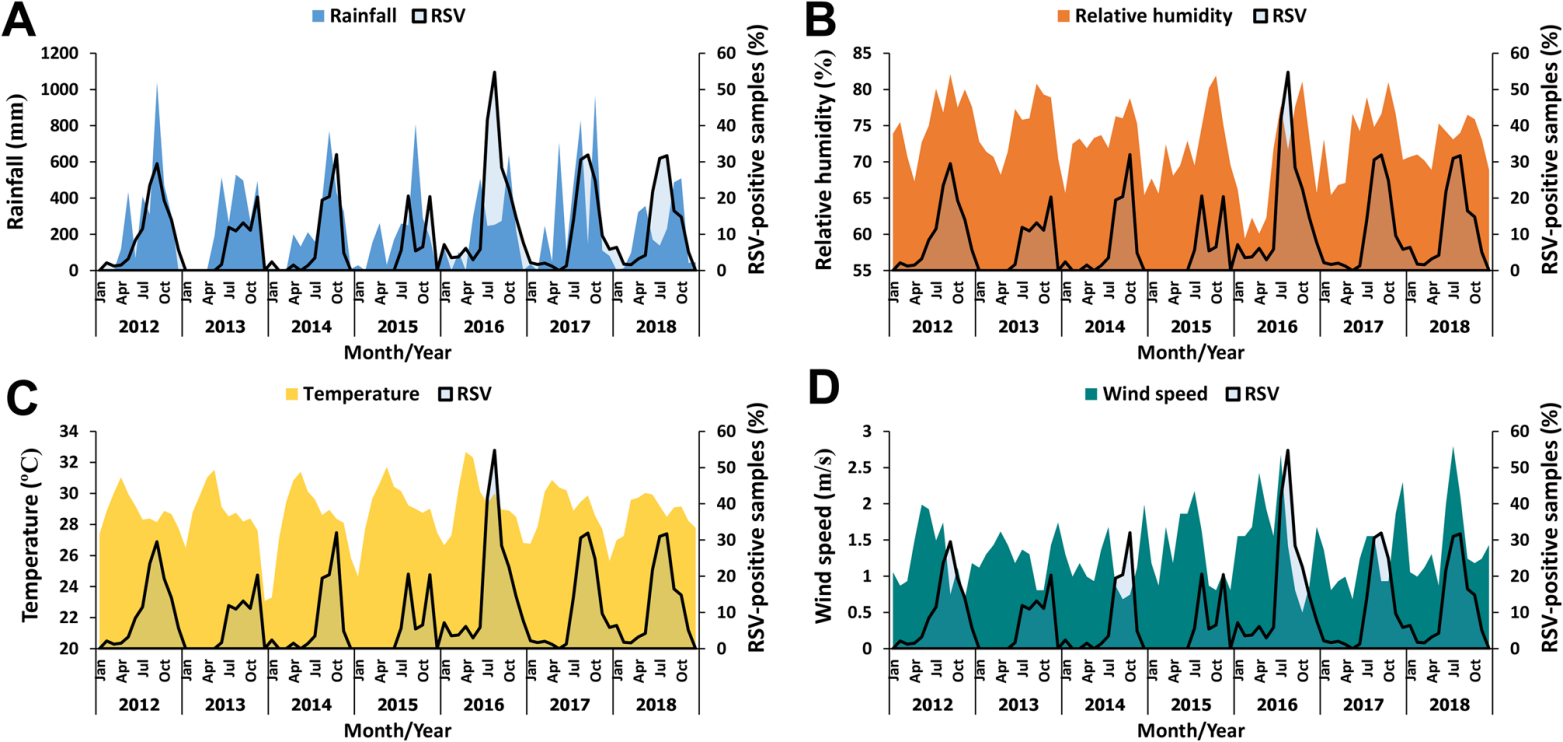
Monthly percentage of RSV-positive samples relative to the monthly meteorological variables. Monthly percentage of RSV-positive samples (black line, right Y-axis) is shown together with the monthly (**A**) rainfall (in millimeters, dark blue region), (**B**) relative humidity (%, brown region), (**C**) temperature (in degrees Celsius, yellow region), and (**D**) wind speed (in meters per second, green region). Climate value scales are shown on the left Y-axis.

Since the prevalence of RSV spiked periodically and appeared seasonal, the potential association between increased RSV activity and various climate factors was examined. Annual fluctuations in the RSV prevalence appeared to coincide with the amount of rainfall and relative humidity (Fig. 2). In some years, RSV activity increases with the increasing rainfall and relative humidity with no lag time (lag 0). However, associations between RSV with the wind speed and temperature showed lags of several months. We therefore performed Spearman’s rank correlation test, which suggests that peak RSV prevalence may lag the wettest month of the year by up to two months (lag 1 and lag 2) (Table 1). Interestingly, peak monthly prevalence of RSV may occur as long as four months after the month with the highest average temperature (lag 4). Analysis using partial correlation similarly confirmed the association for most climate factors, although correlation observed between RSV activity and relative humidity over lag 0 was not significant (Table 2).

**Table 1:** Spearman’s rank correlations of confirmed RSV and meteorological factors.

**Variable**	**Lag 0**	**Lag 1**	**Lag 2**	**Lag 3**	**Lag 4**
Rainfall	0.60^∗∗∗^	0.58^∗∗∗^	0.50^∗∗∗^	0.21	0.02
Relative humidity	0.57^∗∗∗^	0.47^∗∗∗^	0.28^∗^	− 0.04	− 0.26^∗^
Temperature	− 0.13	0.08	0.35^∗∗^	0.63^∗∗∗^	0.75^∗∗∗^
Wind speed	− 0.10	0.01	0.25^∗^	0.41^∗∗∗^	0.34^∗∗^

^∗^ < 0.05, ^∗∗^ < 0.01, and ^∗∗∗^ < 0.001. Lag time between the peak of climate factors and RSV activity noted in months.

**Table 2:** Partial correlation test of confirmed RSV and meteorological factors.

**Variable**	**Lag 0**	**Lag 1**	**Lag 2**	**Lag 3**	**Lag 4**
Rainfall	0.41^∗∗∗^	0.38^∗∗^	0.34^∗∗^	0.09	− 0.03
Relative humidity	0.27^∗^	0.22	0.18	0.14	− 0.08
Temperature	− 0.25^∗^	− 0.004	0.28^∗^	0.58^∗∗∗^	0.71^∗∗∗^
Wind speed	0.19	0.25	0.39^∗∗∗^	0.43^∗∗∗^	0.19

^∗^ < 0.05, ^∗∗^ < 0.01, and ^∗∗∗^ < 0.001. Lag time between the peak of climate factors and RSV activity is noted in months.

**Figure 3:**
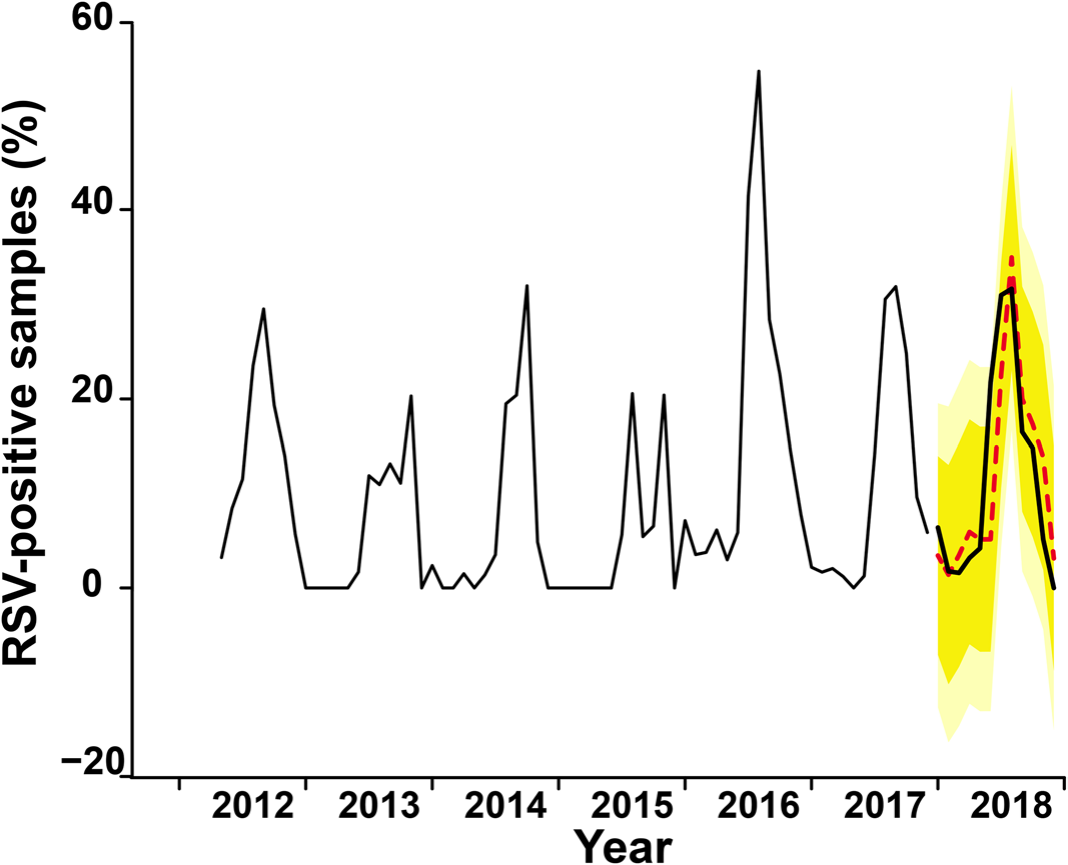
Prediction of RSV incidence for 2018 using 2012–2017 data. RSV cases from 2012 to 2017 (regular line) and 2018 (bold line). Forecast for 2018 from multivariate ARIMA (1,0,0)(2,1,0)_12_ with temperature (lag 4) model is shown (dashed red line) along with the 90% (dark yellow) and 95% (light yellow) confidence interval using R software, version 3.6.1 (https://www.R-project.org).

Assuming that future disease incidence can be predicted based on historical observations, we performed the ARIMA test and fitted the actual observed incidence of RSV with several univariate ARIMA (, , )(, , ) models using different orders. Among the ARIMA univariate models, the ARIMA (1,0,0)(2,1,0)_12_ had the best-fitted performance (Table 3). We next examined several ARIMA models with one or more climate factors at significant lags to find the most appropriate models. From several significant multivariate models, we first tested the ARIMA model for one climate variable as input series. The model with temperature at lag 4 had the smallest AIC and smallest predicted RMSE. We further screened the ARIMA models including two or three climate factors for coefficients presenting the highest significance. For two or three climate factors, no model was found to improve the AIC, fitted or predicted RMSE (data not shown). However, the ARIMA (1,0,0)(2,1,0)_12_ model with temperature at lag 4 appeared most suitable. From January to December 2018, the forecasting of the monthly incidence of RSV with ARIMA (1,0,0)(2,1,0)_12_ utilized temperature data from the previous four months, which yielded the prediction of the expected results for RSV infection (Fig. 3). When considering the lag periods (delays in effect) of 4 months for the influence of temperature on the number of RSV cases, the ARIMAX (1,0,0)(2,1,0)_12_ with temperature forecast model closely resembled the actual observed incidence of RSV for 2018 and was within the relatively large 90% confidence interval.

**Table 3:** Summary of ARIMA model fitting parameters applied with 2012–2017 data.

**Model**	**Fit**		**Pred**	**Climate variables**	
	**RMSE**	**AIC**	**RMSE**	**Lag**	**Coef**
**Univariate ARIMA model**					
ARIMA (1,0,0)(2,1,0)_12_	7.0136	430.34	7.2511	–	–
**Multivariate ARIMA model with the explanatory variables (X)**					
ARIMA (1,0,0)(2,1,0)_12_ with rainfall	6.9820	431.77	7.2052	0	-0.0029
	7.0604	426.27	7.2248	1	0.0011
	6.8234	415.86	7.0386	2	0.0087
ARIMA (1,0,0)(2,1,0)_12_ with relative humidity	7.0016	432.27	7.2018	0	0.0723
	7.0627	426.35	7.2204	1	0.0118
ARIMA (1,0,0)(2,1,0)_12_ with temperature	7.0599	419.74	7.1272	2	− 0.9812
	7.1004	412.73	7.4369	3	1.5326
	**7.1862**	**407.78**	**6.3452**	**4**	**0.9577**
ARIMA (1,0,0)(2,1,0)_12_ with wind speed	7.0302	419.54	7.6797	2	− 2.6356
	6.9719	410.01	6.6576	3	5.5191

*ARIMA* autoregressive integrated moving average with the explanatory variables (X), *fit* fitting results, *RMSE* root mean square error, *AIC* Akaike’s Information Criterion, *Pred* prediction of ARIMA model, *Coef* coefficient of climate variables, *lag* time lag of climate variables. Numbers in bold represent the best model.

## Discussion

Although children get respiratory infections year-round, our multi-year observation of ILI samples testing positive for RSV overwhelmingly predominated in the second half of the year between July and November (mean 18.9%, ± 11.2%) compared to December to June (mean 2.4%, ± 3.6%). The yearly unimodal peak for RSV, particularly around September of 2012 and October of 2013, was also observed in the study conducted by Thailand Ministry of Public Health in conjunction with the U.S. Centers for Disease Control and Prevention^28^. Although both RSV genotypes co-circulate, one dominating genotype in some years is not uncommon. When we examined potential association between peak RSV infection and meteorological data over these seven consecutive years, we observed the strongest correlation of peak RSV detection with certain local climate factors. Highest RSV infection incidence positively correlated with rainfall (r = 0.41, P < 0.001) and relative humidity (r = 0.27, P < 0.05), which were not unexpected given that wet weather naturally increases the humidity. In contrast, RSV incidence negatively correlated with monthly mean temperature (r = − 0.25, P < 0.05). Multivariate analysis using data from the previous six years showed that the best forecasting model was able to trace the observed RSV incidence in 2018, which served as a proof-of-principle for the utility of infection forecast when climate factors especially temperature were considered.

Since there is currently no licensed vaccines nor effective antivirals for RSV infection and it is a major respiratory virus of concern among infants and very young children, continuous surveillance of RSV circulation and the ability to anticipate seasonal upsurge in cases provide a crucial tool in order to mitigate the risk of RSV transmission. Neighboring countries in the region including Myanmar, Laos, and Cambodia share similar climate and socioeconomic factors as Thailand, but are limited in local virological surveillance and laboratory confirmation of RSV in real-time. Thus, accurate forecasting of potential infection annually combined with other measures may be important in limiting the extent of RSV infection requiring hospitalization in resource-constrained economies.

Studies to understand how weather patterns influence RSV seasonality in the tropics have not always been unequivocal. A previous multi-country study showed that the onset of RSV season consistently occurred during the periods of above average rainfall, humidity, and temperature in Thailand, but not in Bangladesh where the weather is also moist and tropical^29^. In the neighboring country of Malaysia, RSV infection peaks in the capital city of Kuala Lumpur between September and December when temperatures are lower than the rest of the year^30^. There, historical prevalence of RSV appears to be associated with rainy days but not absolute rainfall. In contrast to our findings, that study showed that an increased RSV activity was significantly correlated with low relative humidity. The authors suggested that the number of rainy days rather than the absolute amount of rainfall may affect human behavior and thus dictate the risk of respiratory infection. The occurrence of rain but not the amount of rainfall was also associated with the magnitude of RSV infection in a tropical island of Lombok, Indonesia^15^. Similar to our findings, they found a strong correlation between relative humidity and RSV incidence. Although RSV is diagnosed throughout the year in Singaporean children, RSV cases are recorded most in June and July, which does not coincide with their rainiest season^31^. Despite regional proximity and similar climates, epidemiological pattern for RSV infection in Southeast Asia appears to exhibit subtle differences and suggest that climate factors alone do not totally dictate RSV seasonality.

This study has several limitations. Analysis of future trends in RSV incidence alone, although potentially useful, may not be conclusive and is subject to the altering dynamics of societal (political protests, lock-downs, and social distancing) or environmental (such as heat waves, droughts, and massive floods) factors previously encountered in Thailand. It is also difficult to account for human behavior (such as increasing sedentary lifestyle and tendency to congregate in indoor air-conditioned buildings among city-dwellers) or co-circulation of other respiratory viruses in the infection forecast. Furthermore, data on RSV cases under hospital-based settings may not reflect those of the medically non-attended, the community, or the entire country at large. With larger dataset of reliable epidemiological data (regional collaboration comprising of multi-national sites with similar climato-geographical features) and more frequently captured environmental data (hourly or daily instead of monthly), these ARIMA models may be sufficiently refined to provide reliable yearly forecast of RSV onset and peak useful for public health officials in preparing for and to respond to RSV epidemics. Further understanding of the impact of environmental factors on the prevalence of common respiratory viruses can be useful and important in predicting their seasonality.

## Supplementary information

Supplementary file1 (TIF 2248 kb)

Supplementary file2 (PDF 409 kb)

## Data Availability

All data are contained within this manuscript and its supplementary information.
