# Supplementary figures and images for: Respiratory syncytial virus infection trend is associated with meteorological factors
Source: Sci Rep. 2020 Jul 2;10:10931. doi: 10.1038/s41598-020-67969-5 (PMC7331681; doi:10.1038/s41598-020-67969-5)

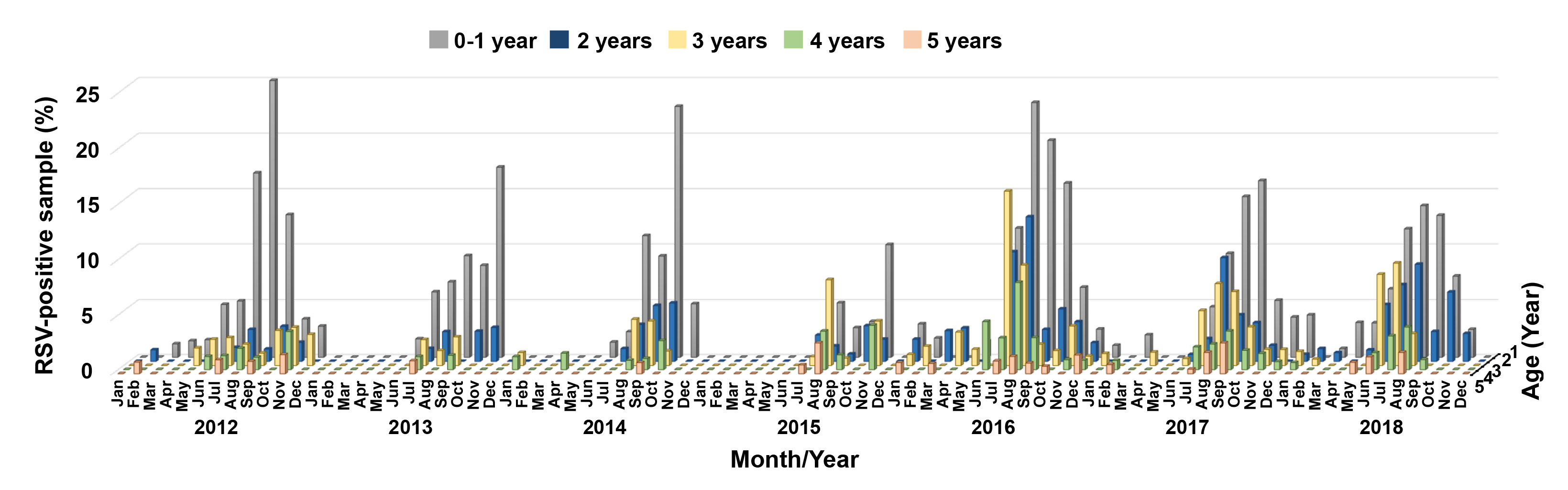

Supplement: Supplementary file 1 — Supplementary file1 (TIF 2248 kb) [file 41598_2020_67969_MOESM1_ESM.tif]
