# Supplementary material for: Respiratory syncytial virus infection trend is associated with meteorological factors
Source: Sci Rep. 2020 Jul 2;10:10931. doi: 10.1038/s41598-020-67969-5 (PMC7331681; doi:10.1038/s41598-020-67969-5)
Supplement: Supplementary file 2 — Supplementary file2 (PDF 409 kb) [file 41598_2020_67969_MOESM2_ESM.pdf]

Respiratory syncytial virus infection trend is associated with meteorological factors

Ilada Thongpan, Sompong Vongpunsawad and Yong Poovorawan

Table S1. The number of samples tested per month for each year during 2012 to 2018.

Year	Month	RSV positive (n)	RSV positive (%)	Total cases (n)
2012	January	0	0.0	26
	February	2	2.1	94
	March	1	1.2	81
	April	1	1.5	66
	May	2	3.2	62
	June	7	8.4	83
	July	9	11.5	78
	August	24	23.5	102
	September	26	29.5	88
	October	12	19.4	62
	November	8	14.0	57
	December	2	5.7	35
2013	January	0	0.0	80
	February	0	0.0	27
	March	0	0.0	61
	April	0	0.0	60
	May	0	0.0	35
	June	1	1.7	59
	July	10	11.9	84
	August	8	11.0	73
	September	10	13.2	76
	October	8	11.1	72
	November	13	20.3	64
	December	0	0.0	16
2014	January	2	2.4	84
	February	0	0.0	43
	March	0	0.0	50
	April	1	1.5	66
	May	0	0.0	65
	June	1	1.4	72
	July	3	3.5	85
	August	23	19.5	118
	September	20	20.4	98
	October	24	32.0	75
	November	2	4.9	41
	December	0	0.0	67
2015	January	0	0.0	76
	February	0	0.0	35
	March	0	0.0	80
	April	0	0.0	67
	May	0	0.0	46

	June	0	0.0	44
	July	7	5.6	124
	August	29	20.6	141
	September	8	5.4	147
	October	4	6.6	61
	November	10	20.4	49
	December	0	0.0	31
2016	January	7	7.1	98
	February	4	3.5	113
	March	4	3.8	106
	April	4	6.2	65
	May	2	3.0	67
	June	4	5.9	68
	July	71	41.5	171
	August	138	54.8	252
	September	58	28.4	204
	October	33	22.6	146
	November	16	14.5	110
	December	9	7.8	116
2017	January	2	2.2	91
	February	2	1.7	119
	March	2	2.1	97
	April	1	1.2	82
	May	0	0.0	79
	June	2	1.3	157
	July	34	14.2	239
	August	78	30.6	255
	September	90	31.9	282
	October	42	24.9	169
	November	13	9.6	135
	December	8	5.9	136
2018	January	10	6.3	160
	February	3	1.7	181
	March	2	1.6	128
	April	3	3.1	97
	May	5	4.7	107
	June	44	20.3	217
	July	44	27.5	160
	August	34	24.6	138
	September	18	14.3	126
	October	13	13.7	95
	November	4	5.1	79
	December	0	0.0	58
	**Total**	**1,082**	**13.2**	**8,209**

Table S2. Data from 2012 to 2018 used for correlation test between climatological parameters and seasonal RSV and for ARIMA model fitting.

Epidemiological_ Month/Year	RSV Positive (%)	Rainfall (mm)	Relative Humidity (%)	Ambient Temperature (°C)	Wind Speed (m/s)
Jan2012	0.0	2.794	73.9	27.37	1.056
Feb2012	2.1	5.842	75.5	28.87	0.8699
Mar2012	1.2	0	70.7	30.03	0.9321
Apr2012	1.5	118.364	67.3	31.04	1.491
May2012	3.2	432.054	72.7	29.96	1.988
Jun2012	8.4	67.056	75	29.2	1.926
Jul2012	11.5	409.448	80.1	28.31	1.491
Aug2012	23.5	310.642	76.8	28.38	1.74
Sep2012	29.5	1037.844	82.1	28.14	0.7456
Oct2012	19.4	471.424	77.5	28.87	1.118
Nov2012	14.0	308.102	80	28.67	0.6835
Dec2012	5.7	0	77.6	27.76	1.181
Jan2013	0.0	0	72.8	26.49	1.118
Feb2013	0.0	0	71.4	28.81	1.305
Mar2013	0.0	3.048	70.7	29.9	1.429
Apr2013	0.0	7.62	68.2	31.04	1.616
May2013	0.0	191.008	71.4	31.52	1.429
Jun2013	1.7	513.588	77.3	29.12	1.181
Jul2013	11.9	263.652	75.8	28.52	1.367
Aug2013	11.0	528.828	76	28.76	1.305
Sep2013	13.2	496.824	80.8	28.19	0.8078
Oct2013	11.1	271.526	79.3	28.38	0.8078
Nov2013	20.3	494.792	78.9	27.62	1.429
Dec2013	0.0	0	70.4	23.07	1.74
Jan2014	2.4	0	65.7	23.3	1.305
Feb2014	0.0	0	72.5	27.09	0.9942
Mar2014	0.0	0	73.2	29.42	1.181
Apr2014	1.5	198.882	71.9	30.84	0.9942
May2014	0.0	132.08	73.3	31.39	0.9321
Jun2014	1.4	211.328	73.7	30.13	1.367
Jul2014	3.5	159.004	71.9	29.61	1.678
Aug2014	19.5	392.43	76.3	28.6	0.8699
Sep2014	20.4	768.858	76	28.94	0.6835
Oct2014	32.0	429.26	78.8	28.36	0.7456
Nov2014	4.9	325.882	75.3	28.1	1.118
Dec2014	0.0	7.62	65.4	25.85	1.988
Jan2015	0.0	29.464	67.7	24.65	1.181
Feb2015	0.0	0	65.6	27.71	0.8699
Mar2015	0.0	154.686	72.4	29.67	1.678
Apr2015	0.0	261.62	68.1	30.63	1.181
May2015	0.0	30.734	69.5	31.7	1.864
Jun2015	0.0	170.18	72.9	30.43	1.864
Jul2015	5.6	259.842	69.5	30.15	2.175
Aug2015	20.6	249.936	74.9	29.23	1.616
Sep2015	5.4	806.704	80.2	29.01	0.8699
Oct2015	6.6	289.306	81.9	28.75	0.8078
Nov2015	20.4	181.61	75.1	29.03	1.056

Dec2015	0.0	0	69.5	27.47	0.8078
Jan2016	7.1	123.19	66.2	26.66	1.553
Feb2016	3.5	0	59.4	27.29	1.553
Mar2016	3.8	101.854	62.3	30.28	1.678
Apr2016	6.2	0	60.1	32.67	2.423
May2016	3.0	293.878	62.3	32.31	1.926
Jun2016	5.9	506.476	71.8	30.12	1.553
Jul2016	41.5	246.126	77.8	29.34	2.672
Aug2016	54.8	252.73	71.7	30.01	1.429
Sep2016	28.4	274.32	77.6	28.95	0.8078
Oct2016	22.6	636.524	81.1	28.9	0.4971
Nov2016	14.5	220.218	73.3	28.5	0.8699
Dec2016	7.8	1.778	65.7	26.81	1.678
Jan2017	2.2	34.798	73.1	26.76	1.367
Feb2017	1.7	0	65.4	27.85	0.8078
Mar2017	2.1	246.38	66.8	30.11	0.9321
Apr2017	1.2	50.038	67.1	30.86	0.9942
May2017	0.0	704.596	76.6	30.39	0.6835
Jun2017	1.3	106.934	74.2	30.2	1.243
Jul2017	14.2	481.838	78.9	28.88	1.553
Aug2017	30.6	828.294	74.8	29.46	1.553
Sep2017	31.9	145.034	76.6	29.87	0.9321
Oct2017	24.9	963.168	81	28.57	0.9321
Nov2017	9.6	111.506	76.6	27.7	1.864
Dec2017	5.9	81.28	70.3	25.63	2.299
Jan2018	6.3	1.27	70.7	26.98	1.056
Feb2018	1.7	13.208	71	27.26	0.9942
Mar2018	1.6	97.79	70.2	29.59	1.118
Apr2018	3.1	321.818	68.9	29.72	1.305
May2018	4.7	356.616	75.3	30.03	0.8699
Jun2018	20.3	170.942	74.2	29.92	1.988
Jul2018	27.5	137.668	73.1	29.19	2.796
Aug2018	24.6	231.14	74	28.49	2.113
Sep2018	14.3	487.426	76.5	29.1	1.243
Oct2018	13.7	508.508	75.9	29.16	1.181
Nov2018	5.1	41.656	73.1	28.24	1.243
Dec2018	0.0	46.228	68.9	27.78	1.429

Table S3. Children who experienced multiple RSV infections in this study.

Child ID	Sex	Year of collection	Month of collection	Age (Year)	Time between infections (Months)
**1**	F	2015	Aug	4	13
		2016	Sep	5	
**2**	M	2015	Aug	3	25
		2017	Sep	5	
**3**	F	2016	Jul	3	14
		2017	Sep	4	
**4**	M	2016	Jul	3	14
		2017	Sep	4	
**5**	F	2017	Jan	3	8
		2017	Sep	4	
**6**	M	2014	Aug	1	23
		2016	Jul	3	
**7**	M	2016	Aug	3	14
		2017	Oct	4	
**8**	F	2014	Sep	1	35
		2017	Aug	4	
**9**	M	2017	Sep	4	13
		2018	Oct	5	
**10**	M	2014	Aug	7 mo	26
		2016	Nov	2	
**11**	M	2016	Aug	2	12
		2017	Aug	3	
**12**	M	2017	Aug	3	4
		2017	Dec	3	
**13**	F	2015	Jul	1	15
		2016	Nov	2	
**14**	M	2017	Oct	3	9
		2018	Jul	4	
**15**	M	2016	Nov	3	11
		2017	Oct	4	
**16**	M	2016	Aug	2	11
		2017	Jul	3	
**17**	M	2016	Sep	1	11
		2017	Aug	2	
**18**	F	2017	Jul	2	12
		2018	Jul	3	
**19**	M	2015	Jul	2	12
		2016	Jul	3	
**20**	M	2016	Sep	1	14
		2017	Nov	2	
**21**	M	2016	Aug	2	11

		2017	Jul	3	
**22**	M	2015	Aug	3	12
		2016	Aug	4	
**23**	M	2016	Aug	1	13
		2017	Sep	2	
**24**	M	2016	Jan	1	7
		2016	Jul	1	
**25**	M	2016	Sep	1	11
		2018	Aug	3	
**26**	F	2016	Aug	3	13
		2017	Sep	4	
**27**	M	2017	Aug	2	11
		2018	Jul	3	
**28**	M	2016	Sep	2	15
		2017	Dec	3	
**29**	M	2016	Aug	1	13
		2017	Sep	2	
**30**	F	2016	Sep	1	11
		2017	Oct	2	
**31**	F	2017	Sep	1	11
		2018	Aug	2	
**32**	M	2016	Jul	6 mo	14
		2017	Sep	1	
**33**	M	2017	Sep	1	10
		2018	Jul	2	
**34**	F	2016	Jul	5 mo	18
		2018	Jan	2	
**35**	F	2016	Mar	2	17
		2017	Aug	3	
**36**	F	2016	Nov	1	20
		2018	Jul	3	
**37**	M	2017	Aug	2	11
		2018	Jul	3	
**38**	F	2016	Aug	9 mo	11
		2017	Jul	1	
		2018	Feb	2	7
**39**	M	2017	Sep	1	10
		2018	Jul	2	
**40**	F	2016	Aug	2	12
		2017	Aug	3	
**41**	M	2016	Aug	5 mo	14
		2017	Oct	1	
**42**	F	2016	Aug	1	13
		2017	Sep	2	
**43**	M	2016	Nov	8 mo	4
		2017	Mar	1	

**44**	M	2016	Sep	1	13
		2017	Oct	2	
**45**	M	2016	Sep	1	12
		2017	Sep	2	
**46**	F	2017	Aug	1	12
		2018	Aug	2	
**47**	F	2017	Oct	3 mo	12
		2018	Oct	1	

F, Female; M, Male; mo, Months.
